# Giant cell arteritis complicated by acute pancreatitis: a case report

**DOI:** 10.1186/1752-1947-2-346

**Published:** 2008-11-17

**Authors:** Deepthi Renuka Seneviratne, Susan P Mollan, Samer Elsherbiny, Theresa Worstmann

**Affiliations:** 1 Birmingham and Midland Eye Centre, City Hospital, Dudley Road, Birmingham, B18 7QU, UK; 2 Queen's Hospital, Belvedere Road, Burton-upon-Trent, Staffordshire, DE13 0RB, UK

## Abstract

**Introduction:**

We describe a case of giant cell arteritis in a woman who was treated with high-dose systemic corticosteroids and subsequently developed acute pancreatitis.

**Case presentation:**

A 78-year-old Caucasian woman presented with four weeks of progressive headache and scalp tenderness. One day before ophthalmology assessment, she had experienced visual obscurations in both eyes. Her visual acuity was 6/9 in both eyes, with a right afferent pupillary defect and right swollen optic nerve. She was diagnosed as having temporal arteritis and was urgently treated with high-dose pulsed intravenous and oral corticosteroids. Her previous diet-controlled diabetes needed insulin and oral hyperglycaemic therapy to control erratic blood sugars. On day 8 of treatment with steroids, she became unwell with epigastric pain and vomiting. She was diagnosed with acute pancreatitis and was treated conservatively.

**Conclusion:**

Acute pancreatitis, a potentially life-threatening condition, is a rare but important side effect of systemic corticosteroids.

## Introduction

Giant cell arteritis (GCA) remains an enigmatic but serious systemic disorder that can lead to total irreversible blindness if not diagnosed and treated swiftly. The recommended treatment is systemic steroids and the initial dose is large [1]. Unlike rheumatologists, ophthalmologists tend to use larger doses, 1.2 to 2 mg/kg per day of prednisolone [1] and this probably reflects the different disease characteristics seen by the two specialities.

The common systemic side effects of prednisolone, such as gastrointestinal disturbances, dyspepsia, weight gain, neuropsychiatric changes and osteoporosis, are well known. However, acute pancreatitis is less well known. Only two cases of acute pancreatitis exist in the ophthalmic literature following high-dose methylprednisolone treatment for acute optic neuritis [2,3]. The authors believe that our case highlights the first reported complication following corticosteroid treatment for vision threatening GCA.

## Case presentation

A 78-year-old Caucasian woman, previously fit and with diet-controlled diabetes, complained of weight loss, progressive malaise, jaw claudication and scalp tenderness for 1 month. One day before assessment, she had had transient complete loss of vision in her right eye and partial loss of vision in her left eye. Her visual acuity was 6/9 in both eyes; colour vision, as tested with Ishihara pseudochromatic plates, was markedly reduced in the right eye (06/17) and normal in the left (17/17). A right afferent pupillary defect was present and dilated fundal examination revealed a right swollen optic nerve. On examination, she had a right tender, nodular, non-pulsatile temporal artery. The erythrocyte sedimentation rate (ESR) was 74 mm/hour (normal for women ≥50 years old: ≤30 mm/hour) and C-reactive protein (C-RP) was >52 mg/litre (normal C-RP <5 mg/litre).

She had emergency treatment with high-dose pulsed intravenous methylprednisolone 250 mg BD and oral prednisolone 80 mg OD to prevent visual loss. She was also started on oral alendronic acid 70 mg once weekly and oral ranitidine 150 mg BD. Her symptoms resolved and her inflammatory markers improved over the next 3 days (ESR 60 mm/hour, C-RP 42 mg/litre), intravenous steroids were stopped and she continued on 80 mg of oral prednisolone OD. However, her glycaemic control worsened (blood glucose 22.8 mmol/litre) and she was managed initially with oral hypoglycaemics (metformin 850 mg OD from day 2); glibenclamide 160 mg BD was added by day 4; and subcutaneous insulin (24 units OM and 6 units nocte) at day 6 finally stabilised the hyperglycaemia (blood glucose 11.6 mmol/litre). At day 8 she became unwell with epigastric pain and vomiting. An abdominal X-ray excluded bowel perforation and clinical chemistry revealed normal liver function tests and an abnormally high serum amylase of 459 U/litre (normal range 0 to 99 U/litre). She was managed conservatively with intravenous fluids and analgesia. Her serum amylase normalised over 48 hours and her symptoms resolved and she was discharged with resolving right disc oedema (ESR 14 mm/hour).

**Figure 1:**
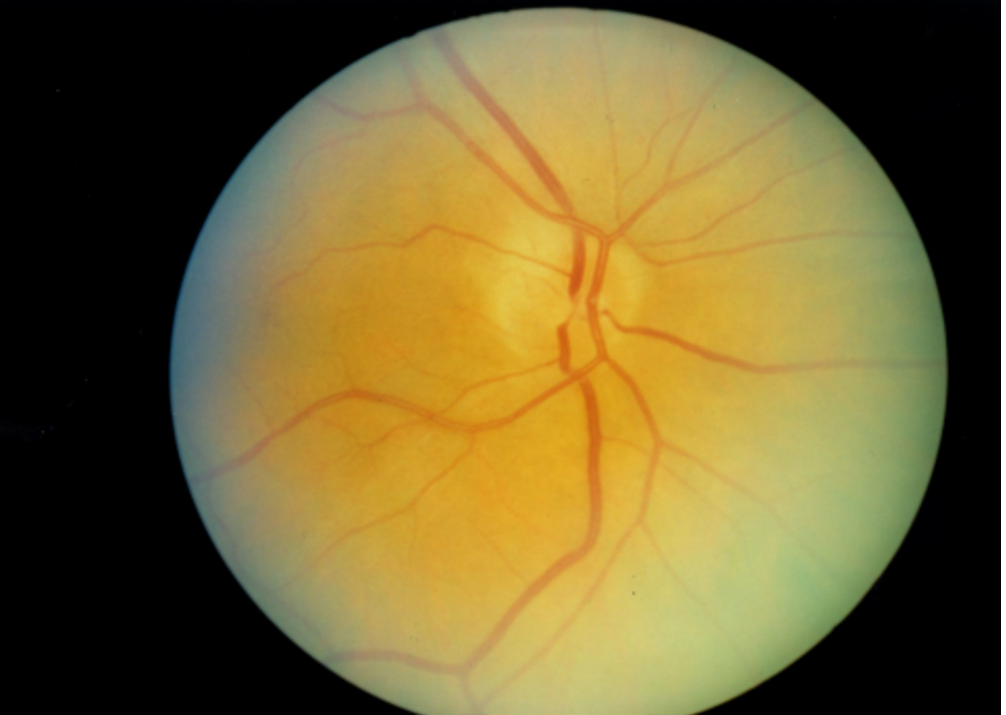
Colour fundus image of the right eye.

**Figure 2:**
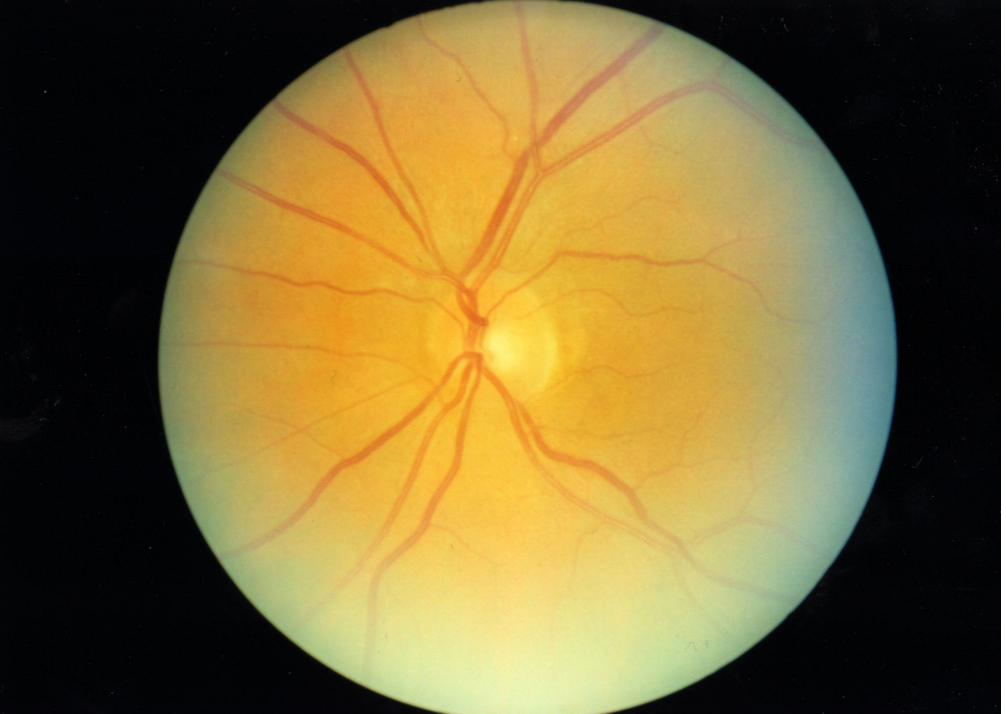
Colour fundus image of the left eye.

She has remained under care with regular blood monitoring and after 8 months is currently maintained on 12.5 mg of prednisolone OD. No symptoms of pancreatitis or GCA have returned. On examination, she has temporal pallor of the right optic nerve (Figure 1) and a normal left optic nerve (Figure 2).

## Discussion

Bowel ischaemia or infarction secondary to involvement of the mesenteric arteries has been reported as a rare extracranial feature of GCA [4]. The blood supply to the pancreas is from the splenic, gastroduodenal and superior mesenteric arteries [5], however, it is unlikely that GCA is the cause in this patient. The pancreatitis started after 8 days of high-dose steroid treatment, there was no evidence of bowel perforation on abdominal X-ray and the pancreatitis settled with conservative treatment and has not reoccurred after 8 months of follow-up. Another possibility is that the pancreatitis could be secondary to disseminated visceral giant cell arteritis. However, this is unlikely as Lie [6], who coined this entity, reported on four male cases none of whom had symptomatic temporal arteritis and all were diagnosed posthumously.

Acid-suppressing drugs, such as H_2_-receptor antagonists and proton pump inhibitors have also been implicated in causing drug-induced acute pancreatitis. A large, UK-based cohort study found no increased risk of acute pancreatitis following acid-suppressing drug administration [7].

Most cases of acute pancreatitis are mild and self-limiting, however, severe attacks can be fatal. Diagnosis is a combination of serum amylase levels three times the normal range (>330 U/litre) and typical clinical features including epigastric pain radiating into the back, nausea and vomiting [8].

Only 1% of acute pancreatitis cases are secondary to systemic steroids, and these cases carry a relatively higher mortality rate (66.7%; n = 4/6) [6] compared to more common aetiological factors such as gallstones or chronic alcoholism [8,9]. The mechanism of corticosteroid-induced pancreatitis is complex and currently unknown.

## Conclusion

It is highly probable that this case of acute pancreatitis was secondary to high-dose systemic steroids and we urge all physicians to be cognisant of this potentially life-threatening side effect.

## Abbreviations

BD: twice a day; C-RP: C-reactive protein; ESR: erythrocyte sedimentation rate; GCA: giant cell arteritis; nocte: at night; OD: once daily; OM: once in the morning

## Consent

Written informed consent was obtained from the patient for publication of this case report and any accompanying images. A copy of the written consent is available for review by the Editor-in-Chief of this journal.

## Competing interests

The authors declare that they have no competing interests.

## Authors' contributions

DS drafted the manuscript. SPM and SE critically reviewed the manuscript. TW supervised the patient management. All authors read and approved the final manuscript.
